# Progress of the Surgery of the Vermiform Appendix

**Published:** 1901-02-02

**Authors:** 


### Progress of the Surgery of the Vermiform Appendix.

**Appendicitis.**—Poynton[1] states that the association of arthritis and appendicitis, though not common, is of interest because it seems likely to throw light upon the pathology of appendicitis. The suggestion that rheumatic fever is an important cause of appendicitis is based upon:—1. The associative occurrence of perityphlitis with acute rheumatism. 2. The occurrence of a polyarthritis resembling that of acute rheumatism coincident with, or shortly after, an attack of perityphlitis. 3. The favourable reaction of some cases of perityphlitis to treatment by salicylates. 4. The similarity in structure of the tonsil and vermiform appendix which, it is suggested, implies a similarity in pathological tendencies. Poynton, however, thinks that the evidence in support of the rheumatic origin of perityphlitis is not conclusive as yet. Of sixty cases of appendicitis which he had investigated there was a history of family or personal rheumatism in eleven, but in none of these could any causal relation between the two diseases be traced. Akerman has shown that intravenous inoculations of the bacillus coli into young rabbits would produce osteomyelitis and effusions into the neighbouring joints, and the bacillus coli had been demonstrated in these metastatic abscesses. In short, Poynton thinks it most probable that the polyarthritis associated with appendicitis is usually of the nature of a pyæmic rather than of a rheumatic lesion, and if this is the case it is an indication for immediate operation. Moullin and Johnson[2] both regard the condition of the pulse as one of the most important indications for or against operations. If the pulse rate at the end of thirty-six hours, while the patient is lying in bed, is over 100 in the minute, or if in the course of the last few hours it has increased in frequency, there is no doubt that the attack is a severe one and that operation will be required. The temperature is no certain guide unless it continues to rise. The intensity of the pain is of great significance, and so are also, but perhaps in less degree, local tenderness, muscular resistance, and a sense of fulness in the right iliac fossa. Robin[3] maintains that a blood count plays an important part in the diagnosis of suppurative appendicitis. The normal proportion of white blood cells is 1 to 300 or 1 to 700 red (5,000–10,000 per cubic millimetre), somewhat higher in women and considerably higher in infants (about 20,000 per cubic millimetre). Physiologically it is increased after a meal, during pregnancy and parturition, after exercise and after a cold bath. Pathological hyperleucocytosis takes place in the following diseases: Hæmorrhage, scarlet fever, diphtheria, tonsillitis, syphilis (secondary), erysipelas, pneumonia, malignant endocarditis, puerperal and other septicæmia (in the case of puerperal septicæmia not of aid in diagnosis), trichinosis, glanders, acute rheumatism, acute multiple neuritis, septic and cerebro-spinal meningitis, infection of the gall bladder, acute pancreatitis, some acute cases of cystitis, gonorrhœa, all kinds of abscess formation, gangrenous inflammations, and various forms of toxæmia. Some drugs (pilocarpine, antipyrin) produce hyperleucocytosis, while others (atropin and its allies) have the opposite effect. Hyperleucocytosis, or diminution in the number of leucocytes, takes place in fasting and malnutrition, and in some infectious diseases, such as typhoid fever, malaria, influenza, measles, mumps, and tuberculosis, except in advanced stages. It is thus seen that a white blood count will often help in the diagnosis of one disease or another. Thus a sudden hyperleucocytosis in the course of typhoid fever will point to a complication, and if accompanied by sudden onset of pain in the abdomen will be sufficient to justify an exploratory incision. A hyperleucocytosis will at once differentiate a suppurative appendicitis from simple colic, typhoid fever, ovarian neuralgia, impaction of fæces, and floating kidney. Developed during the course of a catarrhal appendicitis it will point to suppuration with as much precision as any of the diagnostic signs in our possession. Warden[4] thinks that Doyen's method of removing the appendix is the best. It is simple and rapid; the appendix is not opened, so there is no possibility of infection of the peritoneum by escape of bowel contents; and lastly, by invagination of the stump, in the event of further infection and suppuration the pus is bound to discharge into the lumen of the bowel and to be evacuated naturally per anum. The little mesentery of the appendix is first ligatured with a small silk ligature to free the appendix laterally. Then the following steps are taken:—(1) The base of the appendix is gently crushed with Doyen's small clamp. Almost any forceps suffices for this purpose if strong enough and broad enough to completely occlude the appendix for a breadth of, say, a quarter of an inch. (2) A fine silk ligature is thrown round the base of the appendix in the furrow left by the clamp. (3) The appendix is then removed by the thermo-cautery cutting close to the ligature. (4) A purse suture is then made in the serous covering of the cæcum close round the base of the appendix (as this purse-stitch is drawn tight the little stump is invaginated so that all is completely closed). (5) For safety, a second fine silk purse-stitch is made and the little pucker of the first stitch is similarly invaginated and the ligature is gently tightened. Thiesshaus[5] deals with appendicitis larvata, by which is meant appendicitis of a chronic, insidious character, which moves softly without developing into a real attack of appendicitis. Not infrequently we meet in practice patients complaining of intermittent abdominal pain of varying degrees of intensity, which disappears after a longer or shorter time. On a careful examination of these patients pain may be elicited by palpation, sometimes at McBurney's point, sometimes in the neighbourhood of the navel, or even in the left iliac region or beneath the liver; in some of these cases there are occasionally slight elevations of temperature. In some cases, in which it was believed that movable kidney or retroflexio uteri was the cause of the symptom complex, and in which, in order to overcome these disturbances, the kidney or uterus was fixed, it has been found that these operations had no influence whatever upon the pain, and many operators have had to confess with Wood[6]:—"I can attribute many of my early failures in abdominal work to not directing my attention to the appendix, the pain in the right side persisting as well as the gastro-intestinal disturbances." Whenever the tip of the appendix is adherent to some abdominal organ with or without vascularization of its peritoneal serosa, there is absolute indication for operations. Baldwin[7] mentions two cases of subphrenic abscess following appendicitis. The diagnosis must be based upon the continuance of the symptoms of infection after the removal of the appendix or after the subsidence of the inflammatory symptoms in the region of the appendix. There will also be some tenderness in the region of the liver, with a depression of its lower border owing to the accumulation of pus and exudate between its upper surface and the diaphragm. He recommends an infracostal incision made rather well back on the right side. Thomas[7] reports a case of appendicitis in which pain in the penis was so extreme that the boy was fairly beside himself—rolling and tossing around in a desperate manner, this special symptom overshadowing all others for nearly half an hour. The author has known this same symptom in the perforation of typhoid ulcer. The pain, in such cases, is probably transferred through the hypogastric plexus to the penis.

[1] Lancet, Oct. 27, 1900, p. 1203. [2] Ibid., p. 1204. [3] Med. Rec., Oct. 27 1900, p. 652. [4] Lancet, Aug. 4, 1900. p. 328. [5] Med. Rec., Oct. 6, 1900, p. 531 [6] Amer. Jour. of Obstet., 1899, p. 94. 7. Med. Rec., Oct. 13, 1900, p. 576.

